# 

**Published:** 1894-07

**Authors:** 


### CONTENTS.

COMMUNICATIONS.

Chloroform and Ether ........ 313
Nitrous Oxide ........ 320
Cocaine ........ 334
Local Obtundents ........ 338

SELECTIONS.

Right-Sightedness and Left-Sightedness ........ 356

COMMENCEMENTS.

National University—Dental Department ........ 358
Southern Medical Department—Dental Department ........ 359
Birmingham Dental College ........ 359
University of Pennsylvania—Department of Dentistry ........ 359
College of Dental Surgery of the University of Michigan ........ 360
Columbian University—Dental Department ........ 361
NOTICES ........ 362

EDITORIAL.

The American Dental Association ........ 362
National Association of Dental Faculties ........ 363
Professional Ethics ........ 363
A New Dental College ........ 364
Removal ........ 364
The Southern Dental Association ........ 364

